# Aggregated NETs Sequester and Detoxify Extracellular Histones

**DOI:** 10.3389/fimmu.2019.02176

**Published:** 2019-09-11

**Authors:** Jasmin Knopf, Moritz Leppkes, Georg Schett, Martin Herrmann, Luis E. Muñoz

**Affiliations:** ^1^Department of Medicine 3 – Rheumatology and Immunology, Universitätsklinikum Erlangen, Erlangen, Germany; ^2^Department of Medicine 1 – Gastroenterology, Pneumology and Endocrinology, Universitätsklinikum Erlangen, Erlangen, Germany

**Keywords:** histones, NET formation, aggNETs, proteolysis, autoimmunity, sepsis

## Abstract

In response to various infectious and sterile stimuli neutrophils release chromatin decorated with bactericidal proteins, referred to as NETs. Their scaffolds are formed from chromatin fibers which display an apparent diameter of 15–17 nm and mainly consist from DNA (2 nm) and DNA-associated histones (11 nm). The NET-forming strands are thus not naked DNA but higher ordered chromatin structures. The histones may be released from the NET, especially if their tail arginines have been citrullinated. Several studies indicate that extracellular histones are toxic for mammalian epithelia and endothelia and contribute to the microvascular dysfunction observed e.g., in patients suffering from autoimmune diseases or sepsis. NETs formed at sites of very high neutrophil densities tend to clump and form fairly stable enzymatically active aggregates, referred to as aggNETs. The latter are endowed with a bunch of enzymes that cleave, bind, and/or modify autologous as well as foreign macromolecules. The tight binding of the serine proteases to the matrix precludes the spread of these toxic enzymes into the tissue but still allows the access of soluble inflammatory mediators to the enzymatic active internal surfaces of the NETs where they are degraded. Here, we describe that externally added histones are removed from culture supernatants of aggNETs. We will address the fate of the histones and discuss the feature on the background of neutrophil-driven diseases and the resolution of inflammation.

## Introduction

Histones are a major part of nucleosomes, the basic structural unit of chromatin in the nuclei of eukaryotic cells (1). These nucleosomes each consist of two copies of the histones H2A, H2B, H3, and H4 forming an octamer with 140–150 base pairs of superhelical DNA wrapped around the histone core (2). The linker histone H1 assembles the repeating nucleosome cores into higher-order structures (3). Biosynthesis of histones takes places in the cytoplasm and many histones transiently remain there (4, 5); some reportedly accumulate on the plasma membranes (6). Extra-nuclear localization of histones is also found associated with the DNA structures in neutrophil extracellular traps (NETs), first described in 2004 as bactericidal mechanism (7). Furthermore, histones display anti-microbial activity reviewed in Hoeksema et al. (8), and have been implicated in tissue destruction, sepsis (9), and thrombosis (10). Mechanistically, histones reportedly display direct cytotoxic effect on eukaryotic cells (11), may directly activate phagocytes (12) and platelets (13).

Since their first description in 2004, NETs are now known to play a role in physiology and pathology (14). In high densities these NETs tend to aggregate; these aggNETs, first described to be induced by monosodium urate crystals (MSU) orchestrate the resolution of inflammation in gout by the degradation of inflammatory cytokines (15). With increasing cell densities the proteolytic degradation of cytokines/chemokines outweighs their release (16). The granular neutrophil elastase, a major protein of NETs and aggNETs, degrades various proteins of the extracellular matrix or immunoglobulins (17). Here, we describe that histones are sequestered and detoxified by aggNETs and that this increased viability of epithelial cells in contact to extracellular histones.

## Materials and Methods

### Preparation of AggNETs

We isolated polymorphonuclear cells (PMN) from healthy donors (permit #193/13B from the local ethical committee; written informed consent of participants) by Ficoll density gradient (Lymphoflot, Bio-Rad Laboratories, Inc.) as described previously (18). The granulocytes were then incubated with 50 pg/cell monosodium urate crystals (MSU) for 18 h at 37°C. Successful formation of aggNETs is visible without magnification as depicted in Figure S1E in bright-field as well as under UV (~312 nm excitation) after staining with 1 mg/ml propidium iodide (Sigma-Aldrich) for 2 h. Macrophotographs were taken using a Nikon D700.

### Biotinylation of Histones

We biotinylated calf thymus histones (Sigma-Aldrich) using the EZ-Link™ Sulfo-NHS-LC-Biotinylation Kit (Thermo Fisher Scientific) according to manufacturer's instructions.

### Treatment of Histones With AggNETs/Neutrophil Elastase/Proteinase3

We incubated 1 mg/ml of biotinylated histones with or without (1) aggNETs, (2) 5 mU Neutrophil Elastase (Sigma-Aldrich) or, (3) 5 mU Proteinase3 (Elastin Products Company) in RPMI 1640 medium (Thermo Fisher Scientific) for 24 h at 37°C. If indicated we added the neutrophil elastase inhibitors Sivelestat (6.6 μM) or Elafin (166 μM) (both Sigma-Aldrich).

### SDS-PAGE and Western Blot Analysis

We added 5x PAGE-buffer (2% SDS, 10% glycerol, 5% β-mercaptoethanol, 0.01% bromophenol blue in 60 mM Tris-Cl pH 6.8) to the samples and denatured them at 95°C for 10 min. SDS-PAGE was performed using SERVAGel^TM^ TG PRiME^TM^ 4–20% gels (SERVA Electrophoresis GmbH) for 2.5 h at 100 V. Gels were either transferred onto an Immobilon-P^SQ^ PVDF membrane (Merck Millipore Ltd.) using a Trans-Blot® SD Semi-Dry Transfer Cell (Bio-Rad Laboratories, Inc.) for 1 h at 350 mA or stained with 0.1% Coomassie Brilliant-Blue-G250 (Sigma-Aldrich). Macrophotographs of the Coomassie gels were taken using a Nikon D700. Membranes were blocked with 5% powdered milk (Carl Roth) in Tris-buffered saline (TBS) for 2 h at RT. We detected histone H1 employing rabbit anti-human histone H1.0 antibody [EPR6536] (ab134914, Abcam) overnight at 4°C followed by goat anti-rabbit IgG HRP Antibody (4030-05, Southern Biotech) for 1 h at RT. Biotinylation was detected with Pierce™ High Sensitivity Streptavidin-HRP (21130, Thermo Fisher Scientific). We developed Blots using Celvin® S-320+ (Biostep).

### Prediction and Visualization of Neutrophil Elastase Cleaving Sites

We used the sequence of bovine histone H1.3 (A7MAZ5, UniProtKB) to model its structure with SWISS-MODEL (19). Neutrophil Elastase cleavage sites on histone H1.3 were predicted using the ExPASy PeptideCutter tool (20) and were visualized using the RasMol Molecular Graphics Visualization Tool V2.7.5 (21).

### *In vitro* Histone Cytotoxicity Assay

Analyses by flow cytometry of HeLa cells treated with soluble histones or aggNET pre-treated histones was performed using the four color staining method adapted from Janko et al. (22) and Munoz et al. (23). Briefly, 24 h after seeding of HeLa cells into CELLSTAR® 24-well plates (Greiner Bio-One GmbH), the cells were treated for 1 h with 500 μg of histones, histones pre-incubated with aggNETs or aggNET supernatant in serum-free medium. Mock-treated cells served as controls. After removal of the media to fresh tubes, we washed the cells with DPBS (Thermo Fisher Scientific), detached them using Trypsin/EDTA (Merck) and combined them with the original media. After centrifugation, cells were resuspended in Ringer's solution (Fresenius Kabi) containing 1 μg/ml AnnexinA5, 1 μg/ml propidium iodide, 1 μg/ml Hoechst33342, and 10 nM 1,1′-dimethyl-3,3,3′,3′-tetramethylindodicarbocyanine iodide. Flow cytometry was performed using a Gallios Flow Cytometer (Beckman-Coulter) and Kaluza Analysis Software V2.1 (Beckman-Coulter). Graphs were created using Prism® V5.03 (GraphPad Software). Pictures of cells were taken using a Canon Eos 6D, the Eos Utility3 software (both Canon) in combination with an Axiovert 25 inverted microscope (Carl Zeiss) and the Adobe Photoshop CS5 V12.0.1 (Adobe Systems).

## Results

### AggNETs Proteolytically Degrade Histones

**Figure 1:**
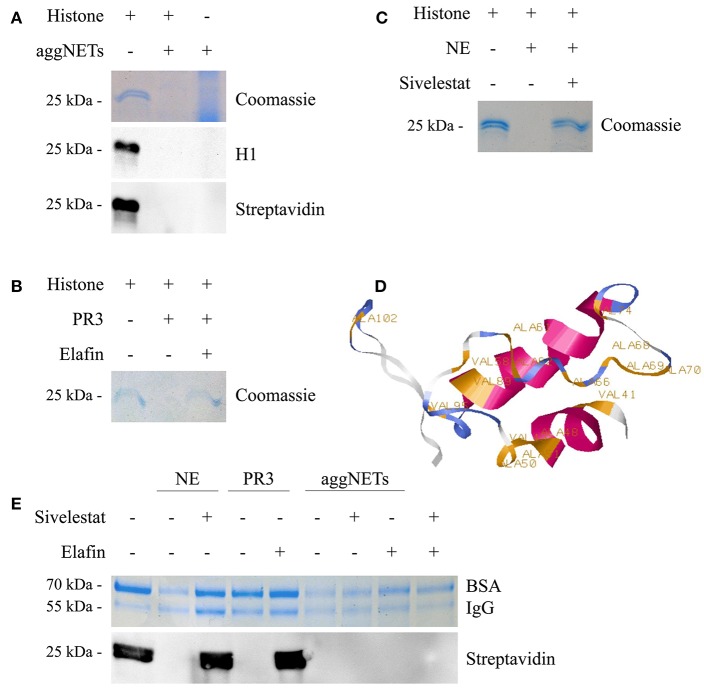
aggNETs degrade histones. **(A)** Histones incubated with aggNETs are degraded as seen in Coomassie and staining and anti-histone H1 Blot. The biotinylationed histones are not bound by the aggNETs. **(B)** Proteinase3 (PR3) degrades histones. This degradation is inhibited by Elafin as seen in the Coomassie staining. **(C)** Neutrophil Elastase (NE) degrades histones, specifically inhibited by Sivelestat as shown in the Coomassie staining. **(D)** SWISS-MODEL of histone H1 (amino acids 39–119) with the cleavage sites for NE as predicted by ExPASy PeptideCutter. **(E)** NE and aggNETs favor histone over bovine serum albumin (BSA) and human Immunoglobulin G (IgG) for degradation; whereas PR3 can only degrade histones. Degradation of biotinylated histones by aggNETs is not inhibited by Sivelestat or Elafin or a combination of both as seen by the detection of Streptavidin HRP in Western Blot analysis. SDS-PAGE, Western Blot Analysis and Coomassie staining in **(A–C)** were performed after incubation of the samples for 24 h at 37°C. For **(E)** the incubation time was 8 h at 37°C. All images shown are representative images of at least three independent experiments. The full-sized images are shown in  Figures S1A–D. The successful formation of an aggNET is shown in the macrophotographs in **Figure S1E** in bright-field and under UV after staining with propidium iodide.

Incubation of calf thymus histones with aggNETs for 24 h results in a complete degradation of histone H1 (Figure 1A) as shown by Coomassie staining of protein. Histone H1 was only detected by Western Blot analysis in the untreated sample, but neither in the aggNET-treated sample nor in the aggNET itself. We biotinylated the histone samples to exclude that the epitope recognized by the antibody was cleaved and therefore not recognized by Western Blotting. The biotinylation was again only detected in the untreated sample but neither in the aggNET-treated ones nor in the aggNETs. Proteinase3 (PR3) and Neutrophil Elastase (NE) are hallmark proteases located in the azurophilic granula of viable neutrophils and on the surfaces of aggNETs. As shown in Figures 1B,C, PR3 and NE degrade histone H1; the reaction is prevented by the specific inhibitors Elafin and Sivelestat, respectively. Prediction by ExPASy PeptideCutter shows that bovine histone H1.3 (amino acids 39–119) exhibits various cleavage sites for NE (Figure 1D). Importantly, this degradation favors histone over bovine serum albumin (BSA) or human immunoglobulin G (IgG) (Figure 1E). Only NE and aggNETs but not PR3 slightly decrease the amount of full-size BSA and IgG. For NE, this was prevented by its specific inhibitor Sivelestat. Surprisingly, neither the addition of Sivelestat nor of Elafin nor a combination of both blocked the degradation of histones by aggNETs at any given time point and concentration.

### AggNET-Treatment of Histones Attenuates Cellular Cytotoxicity

**Figure 2:**
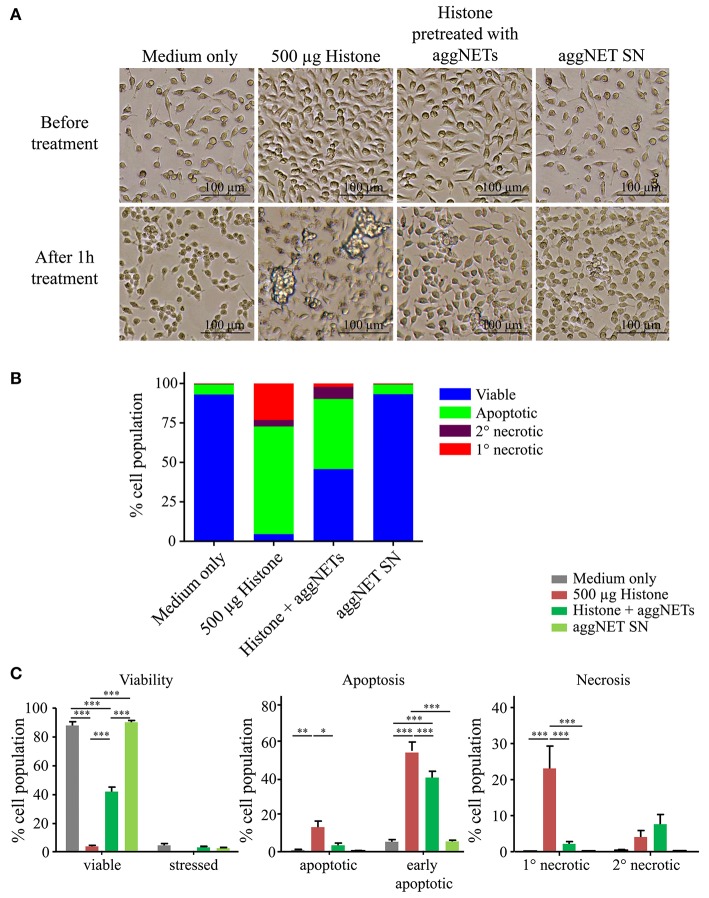
Pre-treatment of histones with aggNETs attenuates histone-mediated cellular cytotoxicity. **(A)** Light microscope images in 10x magnification of HeLa cells before and after treatment with (1) 500 μg histones, (2) histones pre-treated with aggNETs for 24 h or (3) aggNET supernatant (SN). **(B)** Overview of different forms of cell death of HeLa cells after treatment assessed by flow cytometry. Pre-treatment of histones with aggNETs increases viability of HeLa cells. **(C)** Detailed analysis of the different forms of cell death. Viability of HeLa cells incubated with aggNET-treated histones is significantly increased compared to histone treatment due to a reduction in early apoptosis, apoptosis and primary (1°) necrosis. Standard error of mean was calculated from three independent experiments. ***p ≤ 0.001, **p ≤ 0.01, and *p ≤ 0.05 as determined by Two-way ANOVA with Bonferroni post testing. The gating strategy for flow cytometer analysis is depicted in Figure S2.

As soon as 1 h after treatment with 500 μg/ml histone mix HeLa cells are in a bad shape, increase clustering and apparently die as displayed in the bright-field microscopic images (Figure 2A). Pre-treatment of histones with aggNETs prevented this fate. The supernatants of aggNET (aggNET-SN) did not affect the viability of the cells. Flow cytometry revealed that culture in the presence of histones markedly reduced viability and increased apoptosis and necrosis in HeLa cells (Figure 2B). This histone-mediated cytotoxicity is attenuated by pre-treatment with aggNETs. Detailed analyses of the different forms of cell death is depicted in Figure 2C and showed that the pre-treatment with aggNETs significantly decreased early apoptotic, apoptotic and primary necrotic cells; the population of secondary necrotic HeLa cells was only slightly increased. HeLa cells co-cultured with aggNET-SN show comparable viability as medium controls. Therefore, we can exclude that the incomplete rescue in aggNET pre-treated histones is caused by toxic aggNET- derived mediators.

## Discussion

Here, we show for the first time that aggNETs sequester and degrade histones, and thus attenuate their cytotoxic effect on epithelial cells. This process was executed by at least two aggNET-borne serine proteases, NE and PR3. We already have demonstrated the ability of aggNETs to resolve inflammation by the proteolytical degradation of inflammatory cytokines and chemokines (15, 16). NE is established to degrade various proteins, such as immunoglobulins and extracellular matrix components (17, 24). The degradation of histones by NE and PR3 was inhibited by Sivelestat or Elafin, respectively. Importantly, the degradation of histones by aggNETs was resistant to the two inhibitors. Interestingly, a decreased inhibitory capacity of the natural proteinous inhibitors α-1 anti-trypsin and ß2-macroglobulin for membrane-associated NE was already reported before the first description of NET formation (25).

The cytotoxic effect of histones on epithelial cells described here, confirms already existing literature (9, 12). This is especially true when the histone release is exaggerated and not properly controlled. Here, we analyze this cytotoxic effect in more detail using a four color staining method to discriminate between different states of apoptosis and necrosis as described previously (22, 23). The addition of histones to HeLa cells induced profound apoptosis and necrosis (>90% of the cells). This can be partially rescued pre-treating the histones with aggNETs. This procedure increased viability to 50%. To examine if toxic proteins/peptides are released from the aggNETs, we also co-cultured the cells with aggNET-SN only; the cell viability did not differ from medium controls. It is conceivable that some of the small histone-derived peptides, too small to be detected in PAGE, retain residual cytotoxic activities.

Extracellular histones are described as major mediators of death in sepsis due to their contribution to endothelial injury and dysfunction, hemorrhage, thrombosis and organ failure (9). Released histones potentially act as damage-associated molecular pattern molecules (DAMPs) (26) and signal through toll-like receptors (TLR) 2 and 4 leading to a massive pro-inflammatory cytokine production (27). Moreover, histones are shown to enhance plasma thrombin generation and the blood clotting process by involvement of the platelet TLR2 and TLR4 (13). Histones are further released during trauma or severe cellular stress mediating their cytotoxicity by triggering an increased calcium flux in immune and endothelial cells (12).

Histones, released during NET formation, were described in the circulation of patients suffering from autoimmune diseases such as systemic lupus erythematosus (28) or rheumatoid arthritis (29) and were discussed to cause NET-associated tissue destruction. Histones not only act as autoantigens but also prevented the degradation of DNA by the formation of DNA-histone complexes (28). Here we describe that aggNETs degrade and detoxify histones and thus contribute to the resolution of histone-induced inflammatory reactions. If this also takes place *in vivo* and how it can be further enhanced to completely rescue cells from histone-mediated cytotoxicity, needs further investigation. We conclude that histones are targeted by aggNETs for degradation. This leads to a decreased cytotoxicity of histones and, therefore, fosters the resolution of inflammation.

## Ethics Statement

All analyses of human material were performed in full agreement with institutional guidelines and with the approval of the Ethical committee of the Universitätsklinikum Erlangen (permit # 193 13B). Participants gave written informed consent.

## Author Contributions

JK planned and performed experiments, conducted data analysis, and wrote the manuscript. ML, GS, MH, and LM supervised the project, planned experiments, performed data analysis, and wrote the manuscript. All authors read and approved the manuscript.

### Conflict of Interest Statement

The authors declare that the research was conducted in the absence of any commercial or financial relationships that could be construed as a potential conflict of interest.
