# Supplementary material for: Aggregated NETs Sequester and Detoxify Extracellular Histones
Source: Front Immunol. 2019 Sep 11;10:2176. doi: 10.3389/fimmu.2019.02176 (PMC6749074; doi:10.3389/fimmu.2019.02176)
Supplement: Supplementary file 1 [file Data_Sheet_1.pdf]

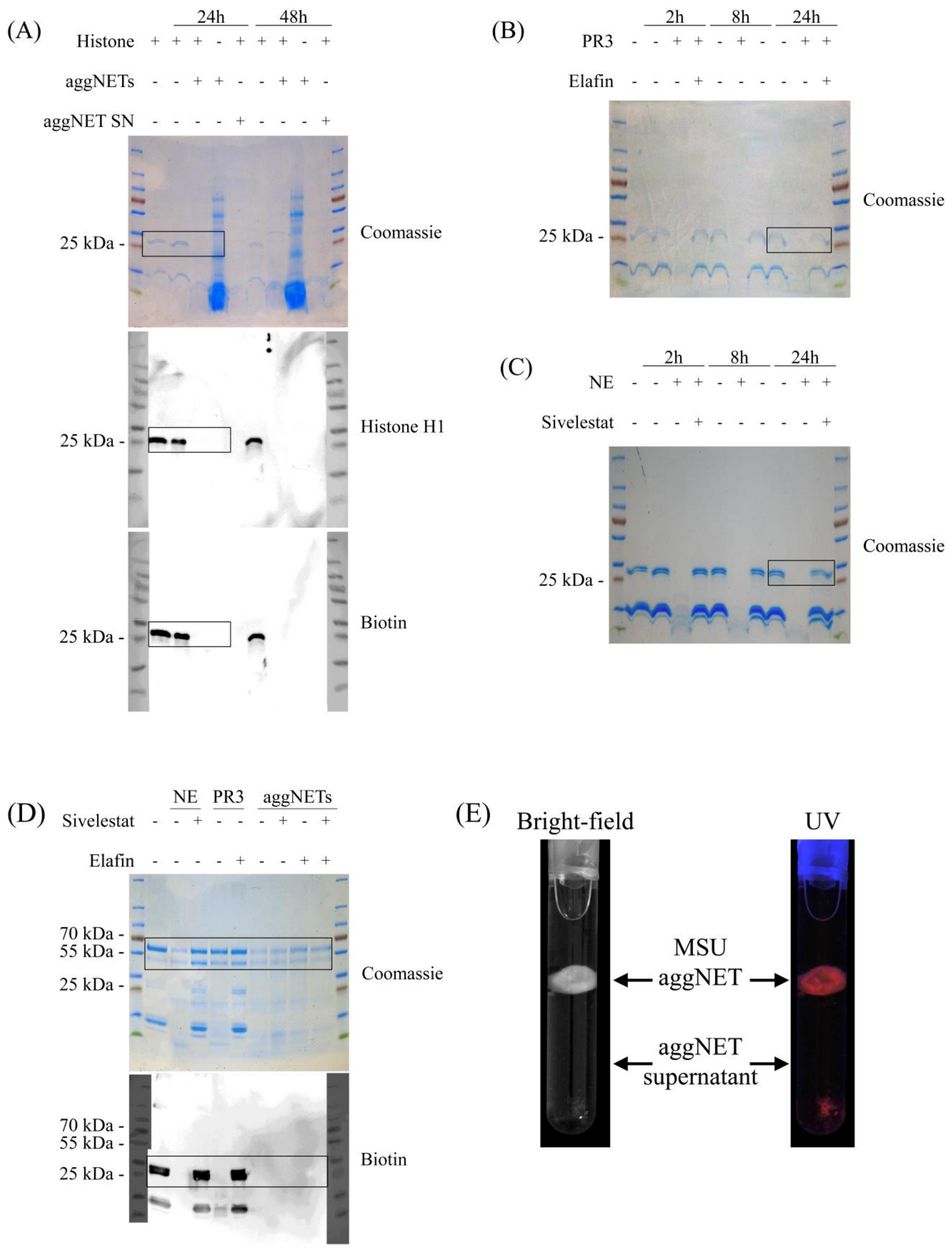

Figure S1 Full-size images of Western Blots and Coomassie gels shown in **Figure 1. (A)** Degradation of Histones by aggNETs. **(B)** Proteinase3 (PR3) is able to degrade Histones. **(C)** Neutrophil Elastase (NE) mediated degradation of Histones is inhibited by Sivelestat. **(D)** NE and aggNETs are also able to degrade bovine serum albumin and human Immunoglobulin G but to a lesser extent. Degradation of histones by aggNETs is not inhibited by Sivelestat and/or Elafin. The protein marker shows a range from 10 – 250 kDa. The black boxes indicate the area that was cut out. (E) Successful formation of an aggNET triggered by monosodium urate crystals (MSU) in bright-field and under UV (~ 312 nm) after staining with 1 mg/ml propidium iodide.

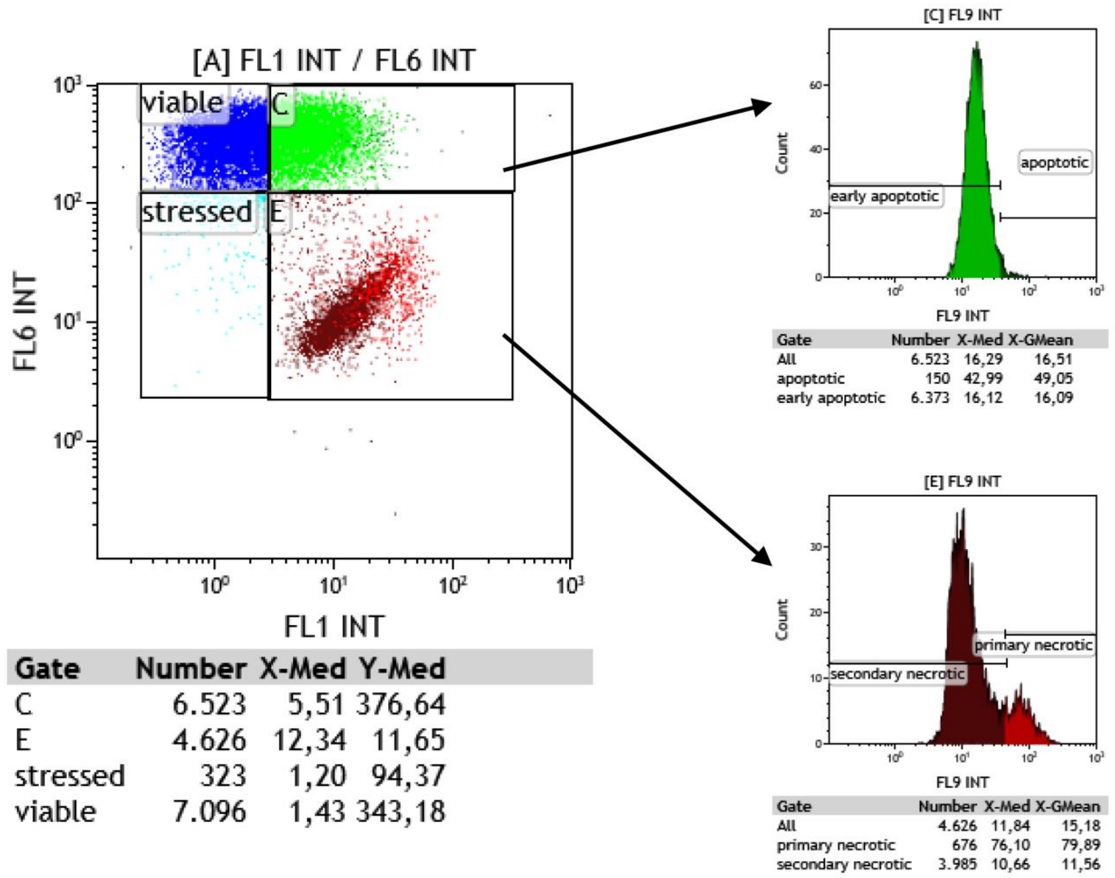

Figure S2 Gating strategy for different types of cell death. Cells were first identified using forward-sideward scatter and then divided into four different populations using 1,1′-dimethyl-3,3,3′,3′-tetramethylindodicarbocyanine, iodide (DilC1(5), Fl6) and AnnexinA5 (Fl1). Populations C and E were then further separated using Hoechst33342 (Fl9) into early apoptotic, apoptotic, secondary and primary necrotic cells.
